# Mechanism of Post-Radiation-Chemical Graft Polymerization of Styrene in Polyethylene

**DOI:** 10.3390/polym13152512

**Published:** 2021-07-30

**Authors:** Anatoly E. Chalykh, Vladimir A. Tverskoy, Ali D. Aliev, Vladimir K. Gerasimov, Uliana V. Nikulova, Valentina Yu. Stepanenko, Ramil R. Khasbiullin

**Affiliations:** 1 Frumkin Institute of Physical Chemistry and Electrochemistry of Russian Academy of Sciences, Leninsky pr. 31-4, 119071 Moscow, Russia; ali_aliev1948@mail.ru (A.D.A.); vladger@mail.ru (V.K.G.); ulianan@rambler.ru (U.V.N.); 4niko7@list.ru (V.Y.S.); khasbiullin@techno-poisk.ru (R.R.K.); 2 Lomonosov Institute of Fine Chemical Technologies, MIREA—Russian Technological University, Vernadsky Avenue 78, 119454 Moscow, Russia; tverskoy@mitht.ru

**Keywords:** post effect radiation graft polymerization, alkoxyradicals, graft polymer distribution profile, morphology, graft polymer structure, degree of crystallinity, graft polymer melting

## Abstract

Structural and morphological features of graft polystyrene (PS) and polyethylene (PE) copolymers produced by post-radiation chemical polymerization have been investigated by methods of X-ray microanalysis, electron microscopy, DSC and wetting angles measurement. The studied samples differed in the degree of graft, iron(II) sulphate content, sizes of PE films and distribution of graft polymer over the polyolefin cross section. It is shown that in all cases sample surfaces are enriched with PS. As the content of graft PS increases, its concentration increases both in the volume and on the surface of the samples. The distinctive feature of the post-radiation graft polymerization is the stepped curves of graft polymer distribution along the matrix cross section. A probable reason for such evolution of the distribution profiles is related to both the distribution of peroxide groups throughout the sample thickness and to the change in the monomer and iron(II) salt diffusion coefficients in the graft polyolefin layer. According to the results of electron microscope investigations and copolymer wettability during graft polymerization, a heterogeneous system is formed both in the sample volume and in the surface layer. It is shown that the melting point, glass transition temperature and degree of crystallinity of the copolymer decreases with the increasing proportion of graft PS. It is suggested that during graft polymerization a process of PE crystallite decomposition (melting) and enrichment of the amorphous phase of graft polymer by fragments of PE macromolecules occurs spontaneously. The driving force of this process is the osmotic pressure exerted by the phase network of crystallites on the growing phase of the graft PS.

## 1. Introduction

Graft polymerization is one of the effective methods for modifying the colloidal–chemical, physical–chemical and performance properties of polymeric materials. Depending on the nature of the monomer, the conditions of graft polymerization and the degree of graft, it is possible to modify the surface layers of polymer material, its volume, and the uniform or gradient distribution of graft polymer over the thickness of a polymer matrix [1,2,3,4].

Radiation graft polymerization allows the introduction of functional groups into polymers of various kinds by the direct graft polymerization of monomers, such as acrylic acid [1,5], vinylpyridines [6,7], acrylonitrile and others [8], or by the subsequent chemical modification of graft polymers [5,9,10]. The latter include PS, the chemical modification of which allows the introduction of a wide range of functional groups into its structure. Radiation graft polymerization on low density polyethylene (LDPE) is the most studied [1,5,8,11,12].

Among various methods of graft polymerization [3], such as “direct” graft, “post effect” graft under irradiation of polymer in a vacuum, inert atmosphere or in air, the “post effect” radiation graft polymerization under irradiation of polymer in air is characterized by its simplicity and high reproducibility. In this case, the polymerization is initiated by alkoxy radicals generated during the decomposition of hydro- and diperoxides. As a rule, salts of variable valence metals are added to the monomer solution as peroxide reducing agents to lower the polymerization temperature. Usually these are iron(II) salts [1,8].

In [9,13,14,15,16], sulfonated membranes obtained by such a method of graft polymerization of styrene on PE film for CO_2_/N_2_, alkane/olefin mixtures separation and proton conducting membranes for fuel cells are used.

However, currently, the studies on influence of graft polymerization parameters on the distribution of graft PS over the cross section of the polymer matrix on PE films of different thicknesses irradiated in air are almost absent. Previously, such studies were carried out only for thin (up to 20 μm) PE films [6,17]. This information is of fundamental importance in the development of technology for the manufacture of asymmetric hybrid membranes for gas separation, namely, when choosing a mathematical model of hybrid membranes.

This work presents the results of an investigation of the effects of the phase structure, morphology and distribution of graft PS in LDPE films up to 200 µm thick on “post effect” radiation graft polymerization in a wide range of graft degrees (composition of graft polymer). The results of the studies of the phase structure of graft PS in LDPE presented in this work allow us to specify more exactly the mechanism of styrene post-polymerization in a polyethylene matrix.

## 2. Experimental

Industrial LDPE films with M_w_ of 49 kDa, M_w_/M_n_ = 11.4 and a refractive index at 130 °C of 1.4386 (NizhnekamskOrgSintez Inc., Nizhnekamsk, Russia) were used as objects of the study. The thickness of the films varied, ranging from 20 to 200 μm, and the size of the films was 2 × 5 cm. The samples were not treated before irradiation. The inhibitor was removed from styrene by 30% aqueous solution of KOH, and then washed with water to pH 7, dried over calcium chloride and distilled twice in a vacuum [1,5].

The PE samples were irradiated using a ^60^Co K-300.500 γ-radiation source at dose rates of 0.0043 Gy/s and from 10 to 100 kGy. The irradiation was performed in air at room temperature. The samples were washed with toluene to constant mass at the end of graft polymerization.

Radiation–chemical graft polymerization of styrene in LDPE samples was carried out by the “post effect” method from a mixture of monomer with methanol. Polymerization was carried out in an open ampoule equipped with a thermoelectric cooler. The process temperature was 80 °C and was due to the boiling point of the solution containing iron(II) sulfate.

As a rule, in this irradiation method, metal salts of variable valence are added as peroxide reducing agents to the monomer solution to reduce the temperature of polymerization [18]. These are usually iron(II) salts [19]. At the end of graft, the films were washed of monomer, homopolymer and iron salts by successive soaking in toluene and methanol and were then air dried to constant mass.

The amount of graft PS (ΔP) was determined by gravimetry as film weight gain (Δm) per unit mass of the original PE film (m_0_) (g of graft PS per mass of PE film):

(1) $$\Delta P=\frac{\left(m_{1}-m_{0}\right)}{m_{0}}\times 100\%.$$

The characteristics of the studied objects are shown in Table 1.

EPR spectra were taken on a standard radio spectrometer RE-1306 (Chernogolovka, Russia).

The distribution of oxygen-containing compounds throughout the PE film thickness, including peroxide formation, was determined using a comparative analysis of the optical density of 1720 cm^−1^ band (C=O valence vibrations in oxidized PE [8]) versus the optical density of 1475 cm^−1^ band (C–H asymmetric deformation vibrations in PE [5]) taken as an internal standard in IR transmission and attenuated total reflection (ATR) spectra. IR spectra were recorded on Perkin–Elmer-580 spectrophotometers (Stockholm, Sweden). ATR spectra were obtained using a KRS-5 crystal with an incidence angle of 45° and a scanning depth of ~5 µm.

Selective contrasting (sulfonation) of graft polystyrene chains was carried out at a temperature of 98 °C with concentrated (96 wt.%) sulfuric acid. At the end of the process, the films were washed with sulphuric acid solutions of decreasing concentrations and distilled water. Sulfonation with a chlorosulfonic acid complex with 1,4-dioxane was carried out in 1,2-dichloroethane at room temperature. The films were then boiled in distilled water to convert the sulphochloride groups to sulphonic acid groups, washed in distilled water and air dried. In all cases, exhaustive sulfonation of phenyl radicals was achieved, the degree of which was monitored by weight gain, similar to the degree of graft. The characteristics of the thus obtained samples are presented in Table 2.

Differential scanning calorimetry (DSC). This method was used to record the thermal effects accompanying the melting (crystallization) and glass transition of the graft copolymer under conditions of programmed temperature change. The heating rate varied from 4 to 50 deg/min. Measurements were performed on a DSC 204 F1 Phoenix (Netzsch, Selb, Germany) in the temperature range from −40 to 150 °C. All experiments were performed on sample weights of at least 5 mg. For automatic processing of measurement results, we used “Proteus Analysis” software, in which the T_g_ value was determined as an inflection point of the ΔC_p_ (T) curve, and the T_m_ value as a maximum (peak) point. The area of the peak, bounded by the DSC curve and the zero baseline, was taken to be equal to the change in melting enthalpy (ΔH_m_). To determine the glass transition temperature, we also used the tangent method [20], which was applied to the left, right and middle parts of the heat capacity step ∆C_p_ (T).

The crystallinity degree of the studied samples was determined from the equation:

(2) $$\alpha =\frac{\Delta H}{\Delta H_{100\%}}$$

where ΔH is the melting enthalpy of the studied sample; ΔH_100%_ is the melting enthalpy for a fully crystalline polymer and ΔH_100%_ = 293 J/g [21].

The structural–morphological characteristics of the graft copolymers were studied by transmission electron microscopy, scanning electron microscopy, and X-ray microanalysis. In the first case, the outer surface of the graft films was used as the study object, which was subjected to etching in high frequency oxygen plasma in order to reveal the supramolecular structure. Oxygen pressure in the etching zone was 0.03 mm Hg, electron energy was 2–3 eV, etching time was 15–20 min, generator power was 100 W, and the frequency was 10 MHz. The morphology of the etched surfaces was examined via a method of one-step carbon-platinum replicas using a transmission electron microscope (TEM-301 (Amsterdam, The Netherlands)) at an accelerating voltage of 80 keV. The phases were identified by X-ray microanalysis using the characteristic Kα radiation of the sulfur line.

**Figure 1:**
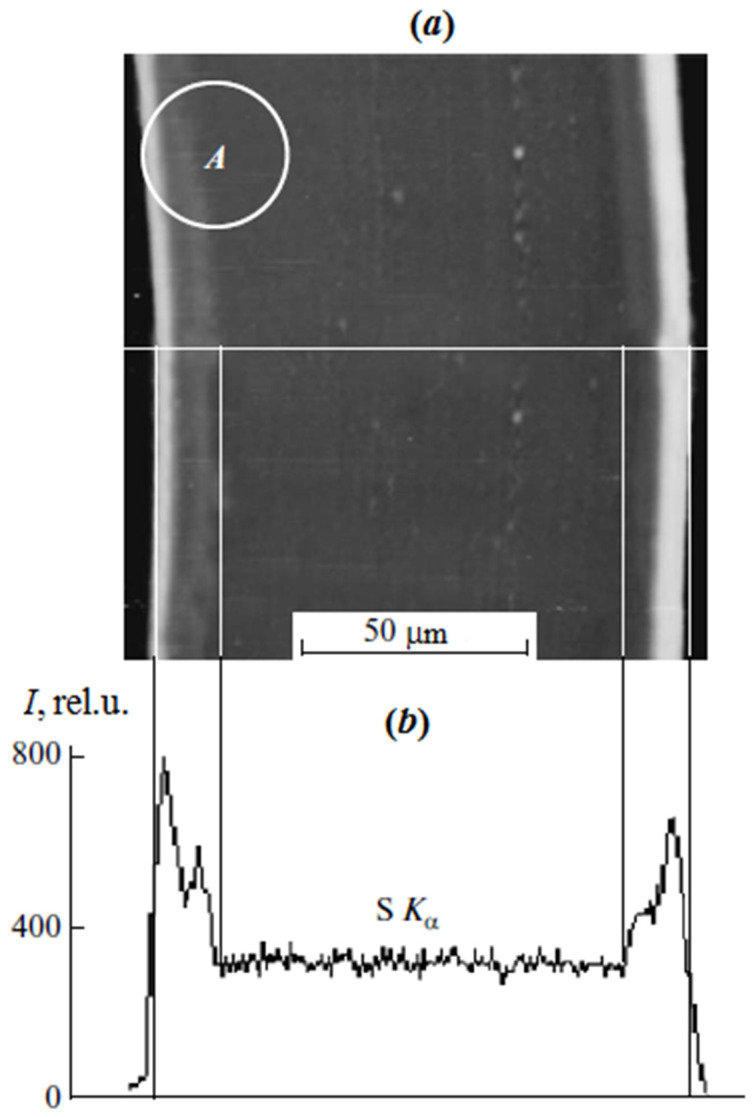
Microphotograph of a cross section of a sulfated PE film with graft PS with ω_PS_ = 0.42 in secondary electrons (**a**) and the profile of the characteristic radiation distribution of the KαS line (contrasted graft PS) along the cross section of the sample (**b**). A is the area where the target replica was obtained.

In the case of scanning microscopy and X-ray microanalysis, the studies were carried out on cross sections of the sulfated PE film with the graft PS (Figure 1) obtained on the LKB cryo ultramicrotome. Surface analysis was performed on a JSM-U3 scanning electron microscope (JEOL, Tokyo, Japan) at an accelerating voltage of 5–7 keV equipped with an energy dispersive spectrometer EUMEX (Hamburg, Germany). The size of the electron beam is 10 nm, the current is 10^−10^ Å and the resolution of the method is 1.3 µm, calculated using the Reed nomogram for contrasted PS samples. These parameters made it possible to obtain information on the cross-sectional distribution of samples of various thicknesses. Figure 1 shows a typical cross section micrograph of a PE film with the graft PS obtained in secondary electrons. It can be seen that the graft layer differs slightly in contrast to the central part. The distribution of the characteristic X-ray emission of the Kα sulfur line shows the change in the PS concentration upon transition from the surface layers of the film to its center.

To measure the surface energy of the graft copolymers (γ_lv_), its polar (γ^P^_lv_) and dispersion components (γ^D^_lv_), the wetting angle measurement method was used, employing a set of liquids with known characteristics (Table 3). A drop of liquid with a volume of 1.10^−3^ to 1.10^−2^ cm^3^ was deposited onto the surface of the polymer sample using a microsyringe. Contact angles were determined on the Easy Drop device (KRUSS, Hamburg, Germany)). All measurements were performed at room temperature. The data obtained by the wetting angle method were processed via the standard instrument software using the well-known Owens and Wendt equation [22,23].

## 3. Results and Discussion

**Figure 2:**
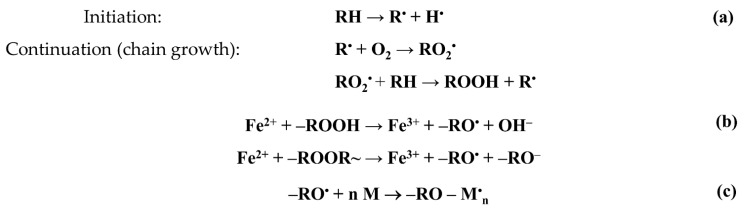
Scheme of radiation–chemical oxidation of PE (**a**), formation of alkoxide radicals (**b**), and growth of the graft polymer chain (**c**).

It is known [1,2,3,4] that the irradiation of polyolefins in air is accompanied by their radiation–chemical oxidation. Radiation oxidation is a chain process and is described by the following scheme (Figure 2).

The process of graft polymerization can be initiated by both trapped hydrocarbon radicals and peroxide radicals and hydroperoxides. In our case, the polymerization was initiated by alkoxy radicals formed during the decomposition of hydro- and diperoxides (Figure 2b). The concentration of alkoxy radicals initiating graft polymerization depends not only on the concentration of peroxides, but also on the concentration of iron(II) salt, which serves not only as an inhibitor of styrene homopolymerization in the graft solution, but also as a reducing agent of hydro- and diperoxides that decompose in the interaction with the iron(II) salt ions into alkoxy radicals and hydroxide or alkoxy anions.

The content of each of these types of active polymerization centers in the polymer depends on the irradiation dose and dose rate, temperature and oxygen concentration during the irradiation and storage of the polymer, its degree of crystallinity, thickness, and other factors. Studies [6] of the EPR spectra of 20 µm-thick PE films irradiated by an electron beam to a dose of 50 kGy showed that peroxide radicals are mainly present in such thin samples, and the appearance and shape of these spectra [7] are virtually the same for all irradiation doses. One hour after irradiation, the concentration of trapped peroxyradicals in the film is 0.4 × 10^19^ spin/g and changes little thereafter. Thus, it was found that after 1 month of storage it was 0.1 × 10^19^ spin/g.

The polymer peroxides formed as a result of radiation oxidation are quite stable, their concentration remains unchanged during film storage, which was confirmed by the reproducibility of the results on styrene graft onto these films over 4 months. The degree of graft PS varied in the range of 135 ÷ 140%.

We studied the distribution of oxygen-containing compounds, including peroxides, over the thickness of PE films modified by irradiation using the ratio of the values of the optical density of the 1720 cm^−1^ band (C=O valence vibrations in oxidized PE [8]) and the 1475 cm^−1^ band (C–H asymmetric strain vibrations in PE [5]), taken as an internal standard in the IR transmission and ATR spectra. It turned out that this ratio calculated from the ATR spectra is 1.7 times higher than that calculated from the transmission spectra. Because the ATR method examines the surface, and the transmission method examines the entire volume of the sample, then we can talk about a significant predominance of peroxide groups in the surface layers.

A similar result follows from a comparison of the PE water wetting angles of 102° before irradiation and 87° after irradiation and the polar component of 0.57 (before) and 3.4 (after) mJ/m^2^. The lower value of the contact angle and the increased value of the polar component unambiguously testify to the polarity of the modified PE surface. Thus, it can be stated that, as a result of irradiation, the surface of PE samples is enriched with oxygen-containing compounds, including peroxides [24], to a greater extent as compared to their volume. We found that the radiation yield of oxygen-containing compounds slightly decreases with increasing sample thickness over 100 μm, which should be taken into account when analyzing the products of graft polymerization [25,26].

**Figure 3:**
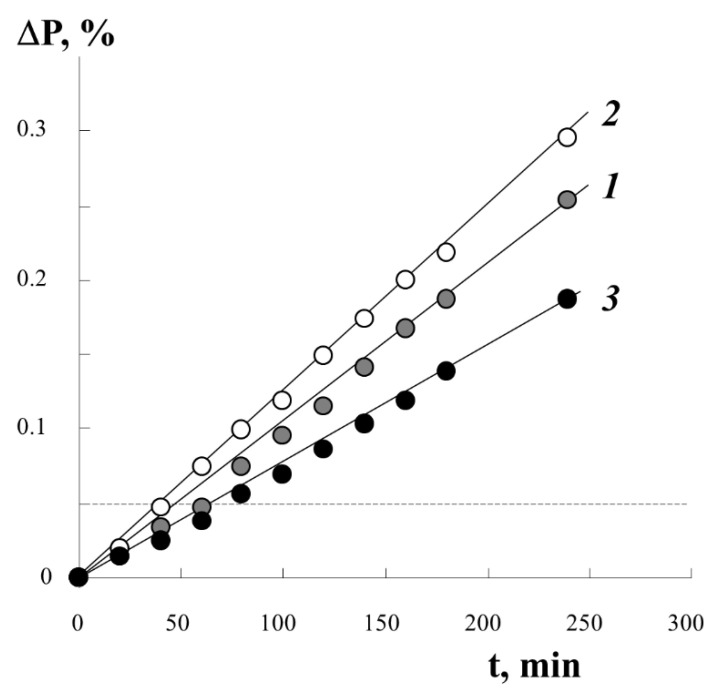
Kinetic curves of styrene graft onto PE under different conditions of the process. Results of gravimetric integral measurements. [FeSO_4_·7H_2_O] content of 2 (**1**), 3 (**2**), 10 (**3**) g/L. Film thickness 140 µm, radiation dose 0.14 Gy.

Figure 3 shows typical kinetic curves of styrene graft onto PE under different conditions of the process. It was found that the irradiation dose D in the studied range of D values from 10 to 250 kGy has little effect on the course of the graft polymerization process. The introduction of different amounts of iron(II) sulfate into the reaction system in the concentration range from 3 to 10 g/L only changes the graft rate. At higher concentrations of iron(II) sulfate, the graft rate decreases by 20–25%. The extreme dependence of the initial polymerization rate on the concentration of iron(II) salt at a constant irradiation dose is related to the fact that iron(II) ions are not only co-initiators, reducing peroxides to alkoxy radicals, but also inhibitors of graft polymerization, interacting with primary and growing radicals:

**−RO^•^ + Fe^2+^ → −RO^−^ + Fe^3+^**

**−R^•^ + Fe^2+^ → −R^−^ + Fe^3+^**

Using information on the concentration of peroxyradicals in the PE film ~0.4 × 10^19^ spin/g, we estimated the degree of graft PS under the assumption of predominant monomolecular filling of the graft centers with a monolayer of styrene molecules. The numerical value is plotted with a dotted line (in Figure 3, a line parallel to the *x*-axis) on the real kinetic curve of the graft. It can be seen that, already in the initial part of the process, we can expect the filling of all centers of graft polymerization.

**Figure 4:**
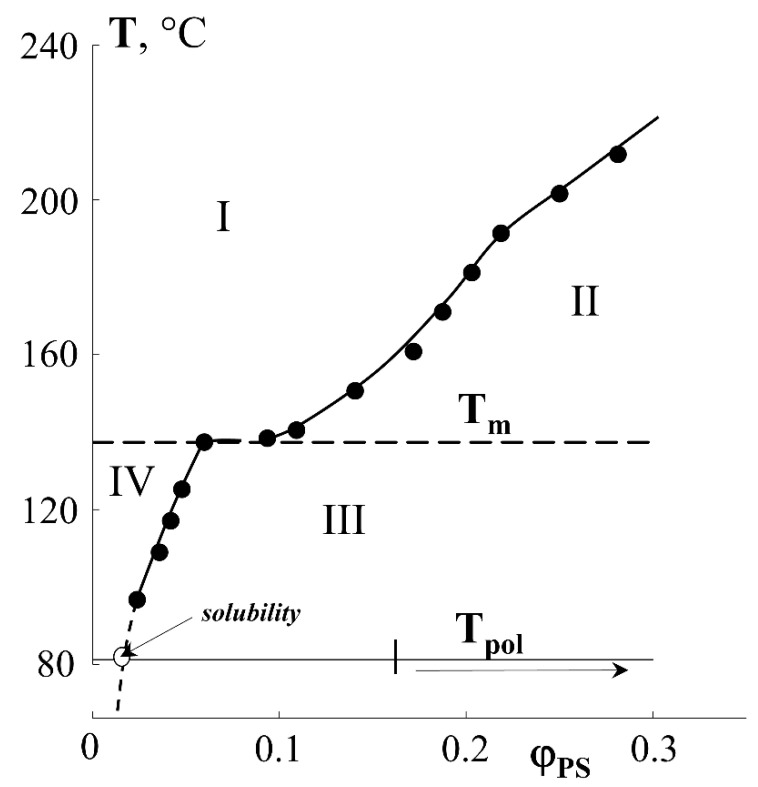
Fragment of the diagram of solubility of PS in PE [27]. The dotted line corresponds to the movement of the system figurative point upon styrene polymerization.

From the macroscopic point of view, in the region of low graft degrees (up to 40 wt.%), the amount of graft PS increases linearly with time. At the same time, no anomalies are observed that would suggest specific features of the structural organization of the graft PS layers in PE films of different thicknesses, despite the fact that the fraction of graft polymer significantly exceeds the solubility limit of PS in PE (Figure 4) [27].

**Figure 5:**
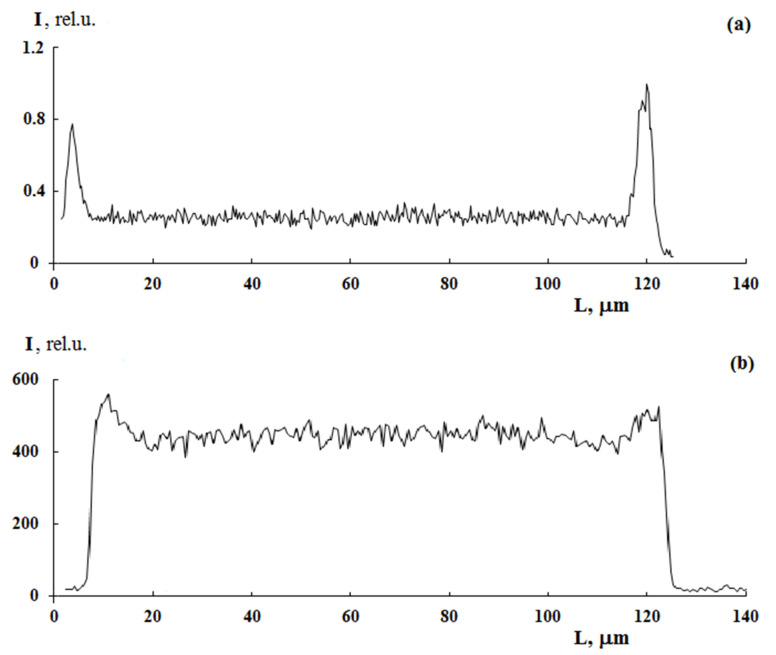
Distribution profiles of graft PS in PE. PE film thickness is 100 µm. Graft degree ΔP = 5 (**a**) and 130 wt.% (**b**). Graft from styrene/methanol mixture (80/20 %vol). Radiation dose −60 kGy. [FeSO_4_·7H_2_O] concentration = 10 g/L.

**Figure 6:**
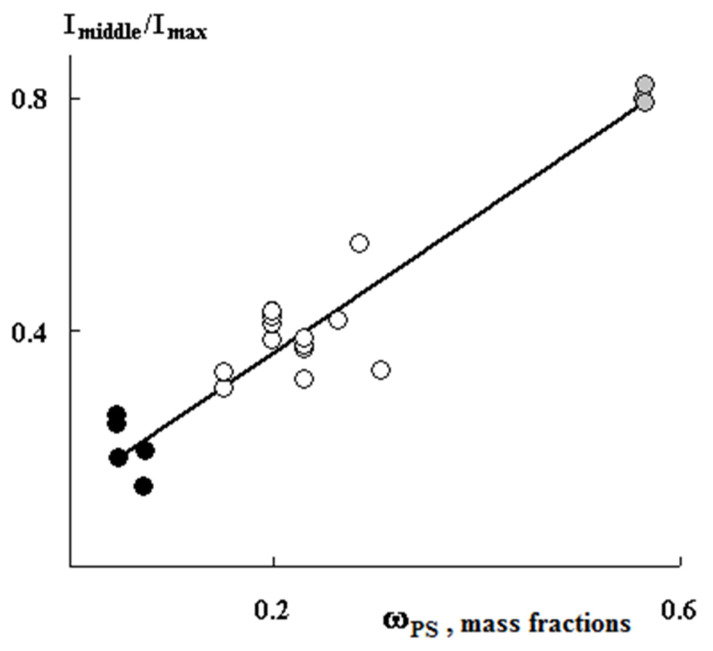
Correlation dependence of the profile intensity in the center of the film relative to maximum intensity on the concentration of graft PS. The thickness of the films is 200 µm (black dots), 20–50 µm (gray dots), 120–150 µm (white dots).

Figure 5 shows PS distribution profiles for samples with different graft degrees obtained under close graft polymerization conditions. It can be seen that, in all cases, the sample surfaces are enriched with PS. At the same time, the PS distribution profiles within the films differ significantly from each other. Thus, at irradiation doses of 10 and 60 kGy, a uniform distribution of PS over the entire sample thickness is realized, that is, a combined three-layer system is formed (Figure 5). Further, as the content of the graft PS increases, its concentration increases both in the volume and on the surface of the samples. A linear correlation between the fraction of graft polymer and the relative concentration of the contrasted polymer is maintained (Figure 6). In our opinion, this result suggests that the mechanisms of graft polymerization on the surface and in the volume of PE samples are identical. In this case, samples of different thicknesses fit into a general linear relationship.

**Figure 7:**
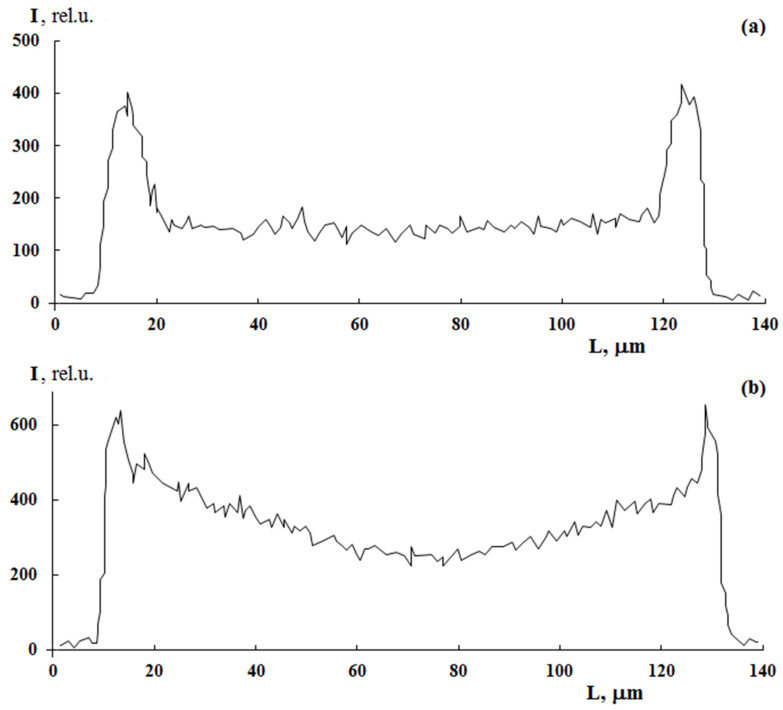
The effect of radiation dose on sulfur distribution profile in PE with graft PS. PE film thickness is 140 µm. Graft from styrene/methanol mixture (70/30 vol.%). [FeSO_4_·7H_2_O] concentration is 3 g/L. ΔP = 30%. Irradiation dose, kGy: 10 (**a**) and 100 (**b**).

A distinctive feature of post effect graft polymerization is, as it seems to us, the stepped form of PS distribution curves, which is preserved up to ΔP = 150 wt.%, on the one hand, and the distribution pattern of the graft polymer in the film volume, on the other hand. At irradiation doses of 100 kGy, the distribution pattern of the graft polymer in the sample volume changes from linear (Figure 7a) to parabolic (Figure 7b). It can be assumed that, under these polymerization conditions, there is a diffusive decline in the concentration of PS in the PE matrix with increased distance from the surface.

A probable reason for this evolution of the distribution profiles of the graft PS may be related both to the distribution of peroxide groups over the sample thickness and to the changes in the translational diffusion coefficients of monomer and iron(II) salt in the graft PS layer and PE matrix. Indeed, as shown above, the surface layers of PE films always possess a higher concentration of peroxide groups, and hence a higher concentration of graft polymer, regardless of the irradiation dose, styrene concentration and iron(II) sulfate concentration. It should be added that methanol is a precipitator of PS. It follows that the growing PS chains have a different conformation on the surface and in the volume of the film. On the film’s surface, they have the conformation of a coiled ball or globule with occluded growing macroradicals. While in the film volume, where the concentration of methanol is much lower, the growing macroradicals probably have the conformation of a swollen ball. In the first case, quadratic polymerization termination is difficult due to the low mobility of the growing PS chains; in contrast, inside the film, where the growing chains have high mobility, quadratic polymerization termination is possible. However, the linear nature of the macroscopic kinetics of graft polymerization allows us to speak about the occlusion of growing macroradicals throughout the volume of the graft polymer, which is associated with its glassy state.

We attribute the presence of the graft polymer concentration jump between the surface and the volume to the high value of the styrene diffusion coefficient in the PE matrix (estimates showed that D ≅ 10^−7^ cm^2^/s), and the near-surface layer, which is ~10 μm thick, does not resist the monomer migration. Thus, it can be assumed that, at this stage, the graft polymerization proceeds in the kinetic region with a constant rate of the process. Since the process is realized under conditions of significant monomer excess, we can assume that the concentration of peroxide radicals is constant, which confirms the above-mentioned assumption about their occlusion by the PS phase. In addition, at this stage of post-polymerization, the transverse dimensions of the samples do not change.

In the region of high graft degrees ΔP ≥ 200%, when the degree of surface and volume filling reaches high values and the size of the near-surface layer reaches 20 μm, the graft process passes into the diffusion region of the process. Note that under these conditions, the translational diffusion coefficient decreases to 10^−10^ cm^2^/s. Probably, a certain contribution to this process is made by the formation of the chemical bonding network in the PE matrix.

**Figure 8:**
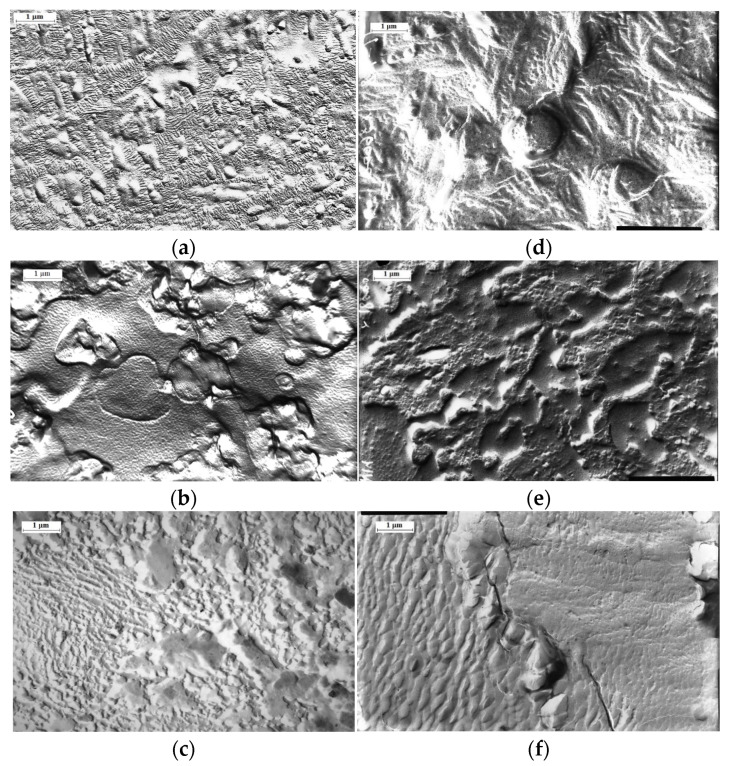
Morphology of the surface (**a**–**c**) and the volume (**d**–**f**) of the samples of graft copolymer. Content of graft PS 16 (**a**,**d**), 47 (**b**,**e**) и 348% (**c**,**f**).

The results of structural–morphological studies are of significant interest for the phase analysis of the system. Thus, according to the results of electron microscope studies, a heterogeneous two-phase system is formed both in the sample volume and in the surface layer during the graft polymerization (Figure 8). It can be seen that the formation of the matrix (PE)-inclusion (PS) type with particle size of 1–3 µm, is observed both in the surface layer (Figure 8a–c) and in the volume (Figure 8d,e) in the area of low graft degrees (ΔP ≤ 5%). As the graft degree increases, the PS particles coalesce to form macroscopically sized aggregates. Finally, at ΔP = 0.4, according to the PS-PE system phase state diagram, the area of phase reversal is observed. It is most vividly manifested in the surface layer of the graft samples.

**Wetting of the graft copolymer surface**. It is known that the wetting test is one of the most sensitive methods for controlling the quality of hydrophobic surfaces and the packing density of graft macromolecules [28]. To describe the wettability of heterogeneous surfaces containing areas of two types, the equation of A. Cassie and S. Baxter [29]:

(3) $$\cos\Theta =f_{1}\cos\Theta_{1}+f_{2}\cos\Theta_{2}$$

(4) $$f_{1}+f_{2}=1,$$

where f_1_ and f_2_ are fractions, occupied by the areas with wetting angles Θ_1_ and Θ_2_.

**Figure 9:**
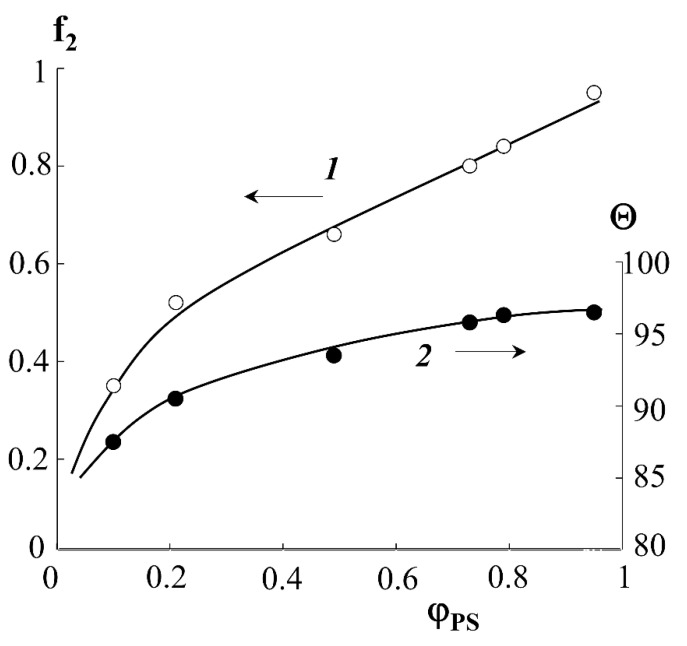
Calculated dependences of the fraction of the graft copolymer surface occupied by PS macromolecules (**1**) and water wetting angles (**2**) on the fraction of graft PS.

Indexes “1” and “2” denote PE and PS phases. Using Equations (3) and (4), as well as the surface wetting data of the graft samples (Figure 9, curve 1), the fraction of the surface occupied by PS molecules depending on the graft degree was estimated. It can be seen that the water wetting angle (sessile) for the hydrophilic–hydrophobic copolymer surface depends on the degree of PS graft and is at the level of 87–100°. The wetting angles plateau at ΔP = 0.6 and Θ ≥ 93°. The fraction of the surface occupied by PS macromolecules increases continuously with increasing degree of graft and reaches 80% for PE-PS samples with ΔP ≥ 0.8.

The results obtained are in good agreement with the data of structural–morphological studies. Indeed, if the curve of the surface area occupied by PS macromolecules is to be plotted onto the phase diagram of the PE-PS system, then using this dependence one can obtain information about the trajectory of the figurative point of the system during graft polymerization. Obviously, at ΔP ≥ 0.4, there is a phase reversal in the graft surface layer and a PS matrix inclusion (PE) type structure emerges, which is described above (Figure 4).

It is interesting to note that the data on the energy characteristics of the graft layers (Table 4)—the dispersion component of the surface energy γ_s_^D^—are in favor of denser packing of PS macromolecules in the region of high graft degrees ΔP ≥ 0.4, which indicates a more perfect conformational order of the macromolecules.

**Figure 10:**
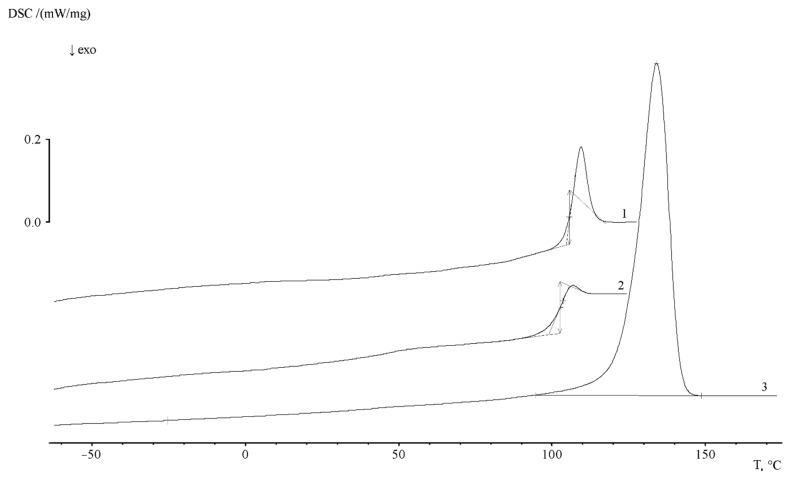
Typical thermograms for PE (**1**) and PS (**2**—first, **3**—second heating) at a heating rate of 20 deg/min.

Additional information about the supramolecular organization of the graft copolymers was obtained by DSC. Figure 10 shows typical thermograms of PE and PS homopolymers. It can be seen that, for PE, as a partially crystalline polymer, there is a melting peak T_m_ and a small bend in the temperature dependence of the heat capacity in the glass transition region T_g_. The heat capacity jump corresponds to ∆C_p_ = 0.041 J/g∙K and a glass transition temperature of −24.5 °C. The beginning and end of melting correspond to 91 and 150 °C, respectively. The melting point of PE is 135.1 °C. Melting enthalpy (∆H_m_, peak area) is 189.8 J/g, the crystallinity degree is 54.8%.

**Figure 11:**
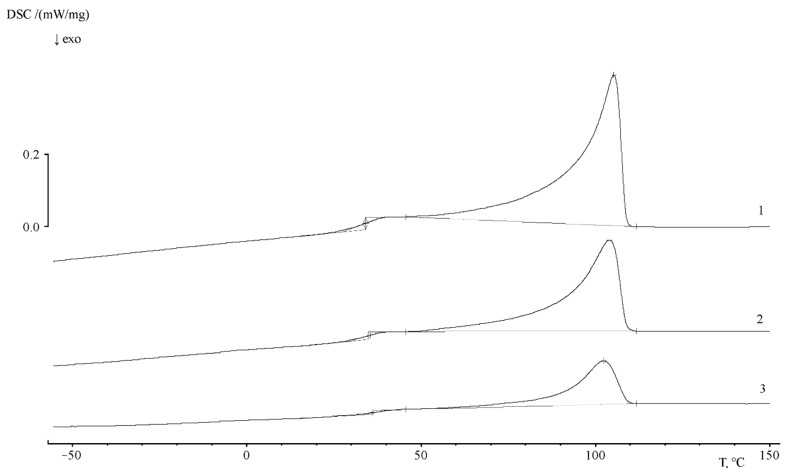
Thermograms of PE/PS samples with ω_PS_ = 0.14 (**1**), 0.49 (**2**) and 0.8 (**3**). Heating rate 5 deg/min.

The heat capacity jump associated with the glass transition of the polymer can be clearly identified in the temperature range of 103 ÷ 119 °C on the thermogram of the first heating of PS. The glass transition temperature of PS corresponds to 107.7 °C. The specific heat capacity jump is 0.329 J/g∙K. The thermogram of the second heating (after thermal annealing) shows that glass transition is observed in the temperature range of 92 ÷ 112 °C, the value of the glass transition temperature corresponds to 104.3 K. The heat capacity jump at this interval is 0.363 J/g∙K. The comparison of the thermograms presented in Figure 11 shows that the region of PS glass transition coincides with the region of the beginning of PE crystallite melting. Obviously, this effect makes it difficult to obtain information about the phase structure of graft copolymers.

The thermograms of the PE–PS16 sample show that, at a heating rate of 5 deg/min, the glass transition is observed in the temperature range of 27 ÷ 40 °C, and the glass transition temperature is 35.2 °C. The heat capacity jump is equal to 0.426 J/g∙K. The melting point of the crystalline part of the copolymer corresponds to 105.4 °C, and the melting enthalpy is 71 J/g. At a rate of 10 deg/min the glass transition interval shifts slightly to a temperature of 22–48 °C, and the glass transition temperature is 47 °C. The heat capacity jump at this interval is 0.472 J/g∙K.

With the increase of graft degree (Figure 11) up to ω_PS_ = 0.49, and then up to 0.8 PS fraction, glass transition is observed in the temperature range of 29 ÷ 50 °C, the glass transition temperature is 42.7 °C, and the heat capacity jump is −0.262 J/g∙K. The melting temperature of the crystalline phase of the copolymer corresponds to 108 °C, the melting enthalpy is 37.33 J/g. The values of melting enthalpy for all samples are presented in Table 5.

**Figure 12:**
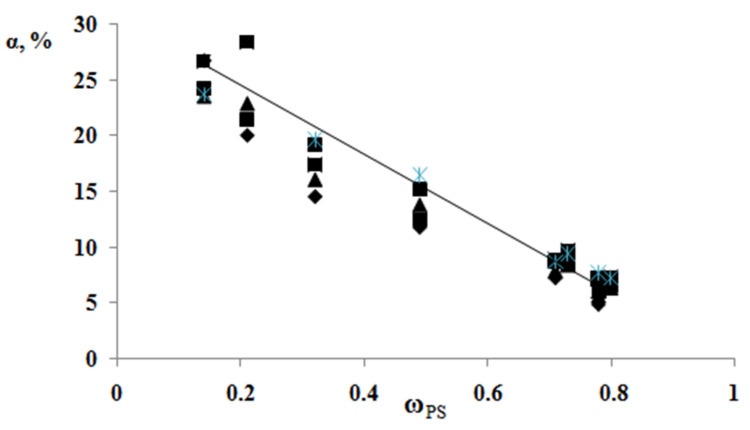
Generalized dependence of the crystallinity degree of the graft copolymer on the PS fragment content.

**Figure 13:**
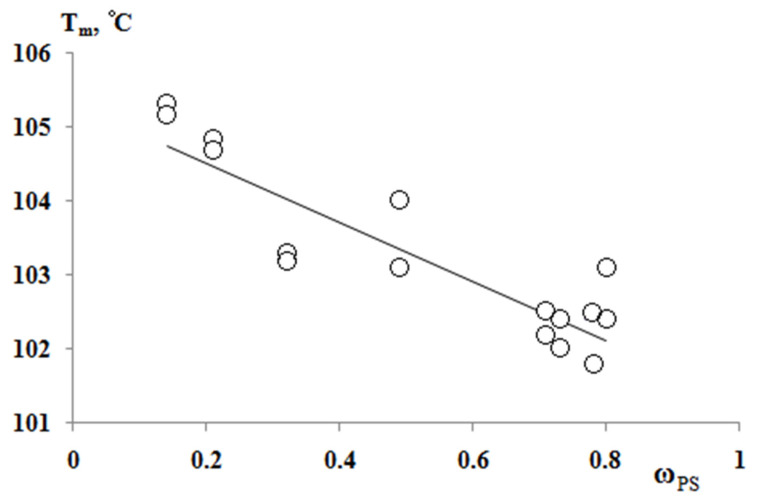
Dependence of the melting temperature of graft copolymers on the content of PS fragments.

**Figure 14:**
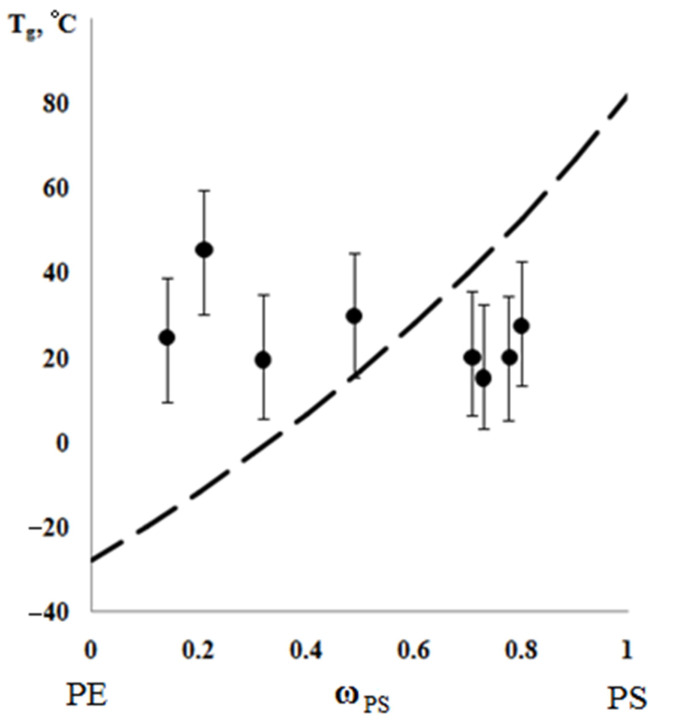
Generalized dependence of glass transition temperature on the composition of graft copolymer. The dashed line is plotted according to the Flory-Fox equation.

According to the results of the thermochemical tests, we obtained unusual information about the effect of the composition of graft copolymer on its degree of crystallinity and glass transition temperature. As an example, Figure 12 and Figure 13 show the dependences of these parameters on the composition of the graft polymer. First, it can be seen that, with an increase in the proportion of graft PS, both the melting temperature of the copolymer and the crystallinity degree of the PE phase decrease. In this case, the greatest effect is observed in the change in the crystallinity degree, less for the melting temperature. Thus, if the PE crystallinity degree in the initial state is ~54%, the crystallinity degree for the graft polymer with 70 wt.% PS content is ~10%, whereas the melting temperature of the graft polymer decreases by only six degrees. Second, it should be noted that the experimentally recorded glass transition temperature of the graft polymer, which characterizes the segmental mobility in the amorphous phase of the graft polymer, practically does not change with changes in its composition in contrast to the glass transition temperature calculated by the Flory-Fox equation (Figure 14). We attribute this effect to the constancy of the composition and the molecular weight of the graft polymer phase.

We assume that the spontaneous destruction (melting) of PE crystallites and the enrichment of the amorphous phase of graft polymer with fragments of its macromolecules occurs in the process of graft polymerization. The driving force of this process is the osmotic pressure exerted by the phase network of crystallites on the growing phase of graft PS. The value of this pressure can probably be estimated from the value of pressure (force) required to expand the sample volume by a degree of swelling and growth of the graft copolymer, which we assume to do in the future.

## 4. Conclusions

Thus, based on the results obtained, we can assume that the formation of the graft PE-PS copolymer involves three processes occurring simultaneously:

(1) The formation of radicals and graft of styrene to the amorphous PE phase;
(2) The formation of radicals and graft of styrene to the PE chains, which are on the surface of the crystallites;
(3) The formation of a heterogeneous PS-PE system with a complex phase organization.

The first process proceeds with a constant composition of the copolymer, which is in the amorphous phase. The second leads to a decrease of the crystallinity degree, and the total degree of graft increases due to this process. The third one is accompanied by the growth of the graft copolymer phase of certain compositions.

## Figures and Tables

**Table 1:** Characteristics of PE films with graft PS.

Sample	Amount of Graft PS (g PS/100 g PE)	Composition of Graft Polymer, PS Mass Fraction (ω_PS_)	Sample	Amount of Graft PS (g PS/100 g PE)	Composition of Graft Polymer, PS Mass Fraction (ω_PS_)
PE/PS16	16	0.14	PE/PS248	248	0.71
PE/PS26	26	0.20	PE/PS271	271	0.73
PE/PS47	47	0.32	PE/PS348	348	0.78
PE/PS96	96	0.49	PE/PS405	405	0.80

**Table 2:** Characteristics of the sulfated PE films with graft PS.

Sample	Amount Graft PS (g PS/100 g PE)	PS Content, Mass Fraction	[–SO_3_H]/[St] g-eq/mol-unit
PE/PS16	16	0.14	0.53
PE/PS47	47	0.32	0.30
PE/PS96	96	0.49	0.16
PE/PS16	16	0.14	1.79
PE/PS47	47	0.32	1.3
PE/PS96	96	0.49	1.5

**Table 3:** Characteristics of the probe liquids [23].

№	Liquid	d, g/cm^3^	T_boil_, °C	γ^P^_lv_, mJ/m^2^	γ^D^_lv_, mJ/m^2^	γ_lv_, mJ/m^2^
1	Water	1.0	100.0	50.2	22.0	72.2
3	Ethyleneglycol	1.1090	197.2	19.0	29.3	48.3
4	o-Tricresyl phosphate	1.165	263.0	4.5	36.2	40.7
5	Pyridine	0.9819	115.4	0.8	37.2	38.0
6	Decane	0.730	174.1	0.0	23.9	23.9

**Table 4:** Energy characteristics of the surface of PE-PS samples with different degrees of graft.

Sample	γ_s_, mJ/m^2^	γ_s_^D^, mJ/m^2^	γ_s_^P^, mJ/m^2^
PE-PS16	30.72	30.15	0.57
PE-PS26	30.31	29.60	0.71
PE-PS96	37.96	37.09	0.88
PE-PS271	41.94	41.23	0.71
PE-PS348	40.69	39.76	0.93

**Table 5:** Melting enthalpy (ΔH_m_) values for all samples.

Sample	Heating Rate				
	5 deg/min	10 deg/min		20 deg/min	
	1st Heating	1st Heating	2nd Heating	1st Heating	2nd Heating
PE/PS16	71.0	78.41	69.24	78.26	69.59
PE/PS26	62.89	58.76	67.27	83.17	
PE/PS47	56.11	42.62	47.15	51	57.75
PE/PS96	44.58	34.54	40.44	36.7	48.38
PE/PS248	25.38	21.19	23.03	26	25.44
PE/PS271	24.76	25.44	24.48	28.45	27.66
PE/PS348	20.93	14.22	16.43	17.64	22.58
PE/PS405	19.19	21.35	18.42	21.38	21.34

## Data Availability

The data presented in this study are available on request from the corresponding author.
